# Synthesis, Spectroscopic and Toxicity Studies of Titanocene Chelates of Isatin-3-Thiosemicarbazones

**DOI:** 10.1155/BCA.2005.151

**Published:** 2005

**Authors:** Garima Vatsa, O. P. Pandey, S. K. Sengupta

**Affiliations:** Chemistry Department D.D.U. Gorakhpur University Gorakhpur 273009 India

## Abstract

The reactions of bis(cyclopentadienyl)titanium(IV) dichloride with a new class of thiosemicarbazone (LH_2_), derived by condensing isatin with different N(4)-substituted thiosemicarbazides, have been studied and products of type [Cp_2_Ti(L)] have been isolated. On the basis of various physico-chemical and spectral studies, five coordinate structures have been assigned to these derivatives. Toxicity studies of titanocene complexes at tbur different concentrations have been carried out against snail *Lymnaea acuminata*. The effect of most potent compounds on the activity of acetylcholinesterase enzyme, which inhibits the activity of enzyme, possibly by the formation of enzyme-inhibitor complex, was also studied.


### Synthesis, Spectroscopic and Toxicity Studies of Titanocene Chelates of Isatin-3- Thiosemicarbazones

Garima Vatsa, O. P. Pandey and S. K. Sengupta*

*Chemistry Department, D.D.U. Gorakhpur University, Gorakhpur-273009, India*
(E-mail : sengupta2002@yahoo.co.in)

### ABSTRACT

The reactions of bis(cyclopentadienyl)titanium(IV) dichloride with a new class of thiosemicarbazone ($LH_2$), derived by condensing isatin with different N(4)-substituted thiosemicarbazides, have been studied and products of type [$Cp_2Ti(L)$] have been isolated. On the basis of various physico-chemical and spectral studies, five coordinate structures have been assigned to these derivatives. Toxicity studies of titanocene complexes at four different concentrations have been carried out against snail ***Lymnaea acuminata***. The effect of most potent compounds on the activity of acetylcholinesterase enzyme, which inhibits the activity of enzyme, possibly by the formation of enzyme-inhibitor complex, was also studied.

### INTRODUCTION

The potential antitumour, antibacterial, antiviral, fungicidal and antimalarial activities of thiosemicarbazones and their metal complexes have spurred the study of the coordination chemistry of these ligands /1-22/. Heterocyclic thiosemicarbazones exercise their beneficial therapeutic properties in mammalian cells by inhibiting ribonucleolide reductase, a key enzyme in the synthesis of DNA precursors /5-8/. Their ability to provide this inhibitory action is thought to be owing to coordination of iron *via* their N-N-S tridentate ligating system, either by a preformed iron complex binding to the enzyme, or by the free ligand complexing with the iron–charged enzyme. Studies of iron and copper complexes have shown that they can be more active in cell destruction, as well as in the inhibition of DNA synthesis, than the uncomplexed thiosemicarbazones.

Recent developments in the structural nature of metal complexes of heterocyclic thiosemicarbazones, depicted below, are correlated with their biological activities.

$$=\overset{1}{N}-\overset{2}{NH}-\overset{3}{C}(S)-\overset{4}{N}R_2$$

It has been suggested /7,8/ that the stereochemistries and activities of complexes often depend upon the anion of metal salt used, the nature of N(4)-substituents and on groups attached to N(1).

The 3-thiosemicarbazones of isatin have been of interest since 1-methyl isatin-3-thiosemicarbazone was found to be active /23-26/ in the treatment of smallpox 40 years ago. Substitution at the N(4) position of the thiosemicarbazone moiety was found to reduce anti smallpox activity. Substitution of two butyl groups at the N(4) position yields a compound with demonstrated activity against *ectromelia* (a vaccina virus) and also against type 2 polio, which is an entrovirus and quite related to the vaccina family. Though biological activity of isatin thiosemicarbazones has been studied, there is little published information /27-30/ on the coordination behavior of this type of ligand. West *et al.* published /26/ a paper on copper (II) complexes with N(4)-substituted isatin-3-thiosemicarbazones. The present paper describes the synthesis, characterization and molluscicidal activity of bis(cyclopentadienyl)titanium(IV) derivatives with isatin-3- thiosemicarbazones.

The structure of the ligands is shown below (I):

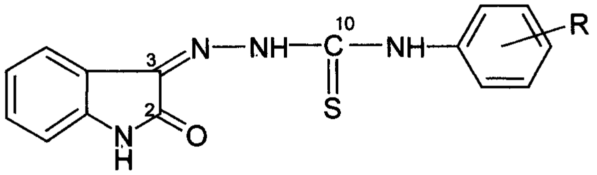

(I)

R=H ($IPTH_2$), 2-$CH_3$ ($IOMTH_2$), 4-$CH_3$ ($IPMTH_2$), 2-$OCH_3$ ($IOMETH_2$), 4-$OCH_3$ ($IPMETH_2$)

### EXPERIMENTAL

All reactions were carried out under strictly anhydrous conditions. THF was dried by heating under reflux over Na wire. The $Et_3N$ was purified by published methods /31/. Bis(cyclopentadienyl)titanium(IV) dichloride was prepared by treating CpNa with the appropriate metal chloride in a $N_2$ atmosphere /32/. The ligands were prepared by the usual condensation reactions between isatin and thiosemicarbazide in methanol /26/. The analyses and physical measurements were made as noted earlier /33/.

For the molluscicidal activity, ten experimental snails were kept in glass aquaria containing 3 L of dechlorinated tap water. Snails were exposed to different concentrations of four compounds [for $Cp_2Ti$(IPMT) and $Cp_2Ti$(IPMET), concentration used (w/v) mg/L : 0.01, 0.025, 0.05 and 0.07; for $Cp_2Ti$(IPT) and $Cp_2Ti$(IOMT), concentration used: 0.025, 0.05, 0.07 and 0.09}. Each compound was mixed with nonionic emulsifier and this mixture was used in treatment. Six aquaria were set up for each test group. Control animals were held in similar conditions without treatment. Mortality was recorded every 24 h up to 96 h. Snail mortality was established by the contraction of the body within the shell, no response to a needle probe was taken as evidence of death. LC values, upper and lower confidence limits (LCL and UCL) and slope value were calculated according to the method of the POLO computer program of Russell *et al.* /34/.

For estimation of enzyme activity three sets of experimental aquaria were set up according to the following dose regimen:

Set 1 Control aquaria contained only dechlorinated tap water.

Set 2 40% of $LC_{50}$ of [$Cp_2Ti$(IPMET)]

Set 3 80% of $LC_{50}$ of $[Cp_2Ti(IPMET)]$

Animals were exposed for 24 h. After treatment for 24 h, snails were removed from the aquaria and washed with water and nervous tissue was taken out for the measurement of AChE activity.

### AChE Estimation

The AChE activity was measured according to Ellman *et al.* /35/ as modified by Singh and Agrawal /36/ Nervous tissue (50 mg) around buccal mass was homogenized in one ml of 0.1M phosphate buffer (pH=8.0) for 5 min in an ice bath and centrifuged at 1000 g for 30 min at –4°C. Supernatants were used as an enzyme source. The enzyme activity at 25°C was measured in a 10 mm-path length cuvette, using an incubation mixture consisting of 0.10 ml ($5\times10^{-4}$M) of freshly prepared acetylcholine iodide solution in distilled water, 0.05 ml of the chromogenic agent DTNB (5, 5-dithiobis-2-nitrobenzoate) reagent, 0.05 ml of enzyme containing supernatant and 1.45 ml of 0.1 M phosphate buffer (pH=8.0). The change in optical density at 412 μm (ΔA/412) was monitored for 3 min. The protein estimation was done by the method of Lowry *et al.* /37/. The enzyme activity was expressed at μM "SH" hydrolysed $min^{-1}mg^{-1}$protein. Each experiment was replicated at least 6 times.

### Preparation of complexes

A mixture of $Cp_2TiCl_2$ (30 mmol) and appropriate isatin-3-thiosemicarbazone (30 mmol) was dissolved in dry THF (*ca.* 60 $cm^3$). To the resulting clear solution, $Et_3N$ (60 mmol) was added and the mixture was stirred for *ca.* 6-7 h at room temp. Precipitated $Et_3N.HCl$ was removed by filtration and the volume of the solution was reduced to *ca.* 15 $cm^3$ under reduced pressure. The colored complex so obtained was recrystallised from a THF/petroleum ether (1:1) mixture.

The details of synthesis, yields and elemental analyses of the isolated complexes are given in Table 1.

### RESULTS AND DISCUSSION

A systematic study of the reactions of bis(cyclopentadienyl)titanium(IV) dichloride with isatin-3-thiosemicarbazone (molar ratio 1:1) in anhydrous THF in the presence of $Et_3N$ may be represented by the following equation :

$$Cp_2TiCl_2 + LH_2 + 2Et_3N \xrightarrow{THF} [Cp_2TiL] + 2Et_3N.HCl$$

$[LH_2=IPTH_2, IOMTH_2, IPMTH_2, IOMETH_2$ or $IPMETH_2]$

Complexes of the type $[Cp_2Ti(L)]$ were soluble in $CHCl_3$, DMF, $CH_3OH$ and $PhNO_2$. The electrical conductance measurements show that the complexes are non-electrolytes. Magnetic susceptibility measurements show that they are diamagnetic.

**Table 1**

Reactions of bis(cyclopentadienyl)titanium(iv) dichloride with isatin thiosemicarbazones

| Reactants | Molar ratio | Stirring time | Product Colour, yield (%), $\Lambda_m$ (ohm$^{-1}$ cm$^2$ mol$^{-1}$) | C | H | N | S | Ti |
|---|---|---|---|---|---|---|---|---|
| | | | | Found (Calcd) % | | | | |
| 1. $Cp_2TiCl_2$+$IPTH_2$+$Et_3N$ | 1 : 1 : 2 | 6 | [$Cp_2$Ti(IPT)] | 63.3 | 4.1 | 11.6 | 6.7 | 10.1 |
| | | | Yellow, 62, 2.25 | (63.5) | (4.2) | (11.8) | (6.8) | (10.2) |
| 2. $Cp_2TiCl_2$+$IOMTH_2$+$Et_3N$ | 1 : 1 : 2 | 7 | [$Cp_2$Ti(IOMT)] | 64.1 | 4.3 | 11.4 | 6.5 | 9.8 |
| | | | Yellow, 60, 3.20 | (64.2) | (4.5) | (11.5) | (6.6) | (9.9) |
| 3. $Cp_2TiCl_2$+$IPMTH_2$+$Et_3N$ | 1 : 1 : 2 | 7 | [$Cp_2$Ti(IPMT)] | 64.1 | 4.4 | 11.5 | 6.5 | 9.7 |
| | | | Yellow, 68, 2.48 | (64.2) | (4.5) | (11.5) | (6.6) | (9.9) |
| 4. $Cp_2TiCl_2$+$IOMETH_2$+$Et_3N$ | 1 : 1 : 2 | 6 | [$Cp_2$Ti(IOMET)] | 62.0 | 4.3 | 11.1 | 6.4 | 9.5 |
| | | | Yellow, 65, 3.12 | (62.1) | (4.4) | (11.1) | (6.4) | (9.6) |
| 5. $Cp_2TiCl_2$+$IPMETH_2$+$Et_3N$ | 1 : 1 : 2 | 7 | [$Cp_2$Ti(IPMET)] | 62.0 | 4.3 | 11.0 | 6.3 | 9.6 |
| | | | Yellow, 70, 3.27 | (62.1) | (4.4) | (11.1) | (6.4) | (9.6) |

$IPTH_2$ = Thiosemicarbazone derived from isatin and thiosemicarbazide of aniline
$IOMTH_2$ = Thiosemicarbazone derived from isatin and thiosemicarbazide of *o*-toluidine
$IPMTH_2$ = Thiosemicarbazone derived from isatin and thiosemicarbazide of *p*-toluidine
$IOMETH_2$ = Thiosemicarbazone derived from isatin and thiosemicarbazide of *o*-anisidine
$IPMETH_2$ = Thiosemicarbazone derived from isatin and thiosemicarbazide of *p*-anisidine

**Table 2**

Infrared spectral bands (cm$^{-1}$) of bis(cyclopentadienyl)titanium(IV) derivatives

| Compound | ν(N-H) | ν(C=N) | ν (C-S) | ν (Ti-O) | ν (Ti-N) | ν (Ti-S) | $C_5H_5$ ring |
|---|---|---|---|---|---|---|---|
| [$Cp_2$Ti(IPT)] | 3320m | 1600s,1570s | 610m | 480m | 440m | 380m | 3000w1420m,1000m,800w |
| [$Cp_2$Ti(IOMT)] | 3340m | 1610s,1570s | 600m | 485m | 435m | 370m | 3010w,1425m,1010m,810w |
| [$Cp_2$Ti(IPMT)] | 3310m | 1590s,1565s | 605m | 475m | 440m | 375m | 3000w,1415m,1010m,815w |
| [$Cp_2$Ti(IOMET)] | 3334m | 1595s,1560s | 595m | 480m | 445m | 385m | 3000w,1410m,1015m,800w |
| [$Cp_2$Ti(IPMET)] | 3325m | 1585s,1565s | 600m | 478m | 430m | 378m | 3015w,1425m,1015m,805w |

### Infrared Spectra

The characteristic infrared spectral bands of bis(cyclopentadienyl)titanium(IV) derivatives are given in Table 2. The ligands show bands at *ca.* 3350-3300, 3230-3200 and 1620-1600 $cm^{-1}$, assignable /26/ to υ($N^{(4)}$H), υ($N^{(2)}$H) and υ(C=N), respectively. In the complex, the first band remains almost at the same position, indicating the ($N^4$H) nitrogen atom is not coordinated to the metal. The second band at *ca.* 3230-3200 $cm^{-1}$ is absent in the complexes; however the third band (*ca.* 1620-1600 $cm^{-1}$) is lowered (*ca.* 15-20 $cm^{-1}$) in the complexes, indicating coordination of the azomethine nitrogen to titanium. In the far i.r. spectra, the bands at *ca.* 445-460 $cm^{-1}$ are tentatively assigned /33/ to υ(Ti-N) vibration.

The four bands due to thioamide group vibrations /38,39/ appear at *ca.* 1460-1500, 1270-1280, 1040-1020 and 785-760 $cm^{-1}$ in spectra of ligands. These bands of the ligands, due to the mixed contributions of δ(N-H), υ(C-N), υ(C-S) and δ(C-H) vibrations, are found to be absent in the spectra of the complexes. The disappearance of thioamide bands in the complexes indicates the possibility of thione $\rightleftharpoons$ thiol tautomerism. The i.r. spectra show a new band at *ca.* 600 $cm^{-1}$ owing to conversion of C=S into $C\text{-}S^-$ . The new band in the complexes at *ca.* 370-385 $cm^{-1}$ is assigned to υ(Ti-S), and shows that sulfur is bonded to titanium. In addition, the spectra of the ligands show bands at *ca.* 3180 and 1680 $cm^{-1}$ which are assigned /26/ to υ(N-H) and υ(C=O) vibrations of isatin moiety. These bands disappear in titanium (IV) complexes, which may be due to enolization of keto group. This is further confirmed by the appearance of new bands at *ca.* 1570 and 1525 $cm^{-1}$ assignable /40/ newly formed υ(C=N) and υ(NCO) groups. The coordination through enolic oxygen through deprotonation is confirmed by the appearance of band at *ca.* 480 $cm^{-1}$ assignable /41/ υ(Ti-O) vibration.

Absorption bands occurring at *ca.* 3000 $cm^{-1}$ for υ(C-H), *ca.* 1420 $cm^{-1}$ for υ(C-C), *ca.* 1010 $cm^{-1}$ and 810 $cm^{-1}$ for (C-H out of plane deformation) in the complexes are due to the cyclopentadienyl ring. These bands are similar to those reported for bis(cyclopentadienyl)-titanium (IV) dichloride and their appearance indicate that the ($\eta^5$-Cp) group persists in the complexes.

Thus, the infra-red spectra reveal that isatin-3-thiosemicarbazones behave as dibasic, tridentate ligands coordinating through thiol sulphur, enolic oxygen and azomethine nitrogen.

### $^1$H n.m.r. Spectra

The $^1$H nmr spectra of the [$Cp_2$TiL] type complexes have been recorded in DMSO-$d_6$. (Table 3). Coupling between various groups complicates the spectra, but a comparison of the spectra of ligands with those of the complexes can lead to the following conclusion:

(a) The δ 6.65-6.80 signals may be assigned to the cyclopentadienyl ring protons and indicate the rapid rotation of the ring about the metal ring axis.

(b) The signals of N(2)H and N(4)H are seen at *ca.* δ(9.2) 1H and δ(8.9) 1H, respectively, in the ligands. The spectra of the complexes show the absence of first peak and the presence of second peak almost at the same position.

(c) The chemical shift due to aromatic ring appears at *ca.* 7.8-8.1 ppm, which slightly shifts downfield in the complexes. This may be due to decrease of electron density after forming the complex.

(d) The peak due to N(I)H of isatin ring appears at *ca.* 11.2 ppm in the spectra of ligands which disappear in the corresponding complexes.

**Table 3**

NMR spectral data ( δ scale, ppm) of bis(cyclopentadienyl)titanium(IV)

| Compound | $^1$H NMR | | | | $^{13}$C NMR | | | |
|---|---|---|---|---|---|---|---|---|
| | $\eta^5$- $C_5H_5$ | -NH | Phenyl ring | $-CH_3$ | $\eta^5$-$C_5H_5$ | C(2) | C(3) | C(10) |
| [$Cp_2$Ti(IPT)] | 6.70s | 8.95s | 7.85-7.92m | - | 115.8 | 132.6 | 125.8 | 146.2 |
| [$Cp_2$Ti(IOMT)] | 6.75s | 8.90s | 7.80-7.95m | 2.40s | 116.4 | 138.5 | 128.6 | 148.5 |
| [$Cp_2$Ti(IPMT)] | 6.78s | 8.92s | 7.86t, 8.10t | 2.48s | 116.0 | 136.2 | 126.5 | 147.2 |
| [$Cp_2$Ti(IOMET)] | 6.80s | 8.98s | 7.86-7.98m | 3.42s | 115.5 | 133.8 | 126.0 | 147.0 |
| [$Cp_2$Ti(IPMET)] | 6.65s | 8.96s | 7.82t, 8.05t | 3.45s | 116.2 | 131.6 | 125.9 | 146.8 |

### $^{13}$C n.m.r. Spectra

The $^{13}$C nmr spectra of [$Cp_2$TiL] type complexes were recorded in DMSO-$d_6$. The following are the salient features.

(a) The peak due to cyclopentadienyl groups appears at *ca.* 116 (relative to TMS).

(b) The ligands show amide C(2) and thioamide C(10) at *ca.* δ160 and *ca.* δ150, respectively. In the complexes, these signals are at significantly higher field. The significant shift in the position of C(2) (*ca.* δ 130-135.8 in the ligand) may be due to enolization of the keto group and formation of new azomethine linkage. The signal due to C(3) appears in the region (*ca.* δ 125.8-128.6).

On the basis of elemental analysis, electrical conductance measurements and spectral data, the following structures (II) are tentatively proposed for [$Cp_2$Ti(L)] complexes.

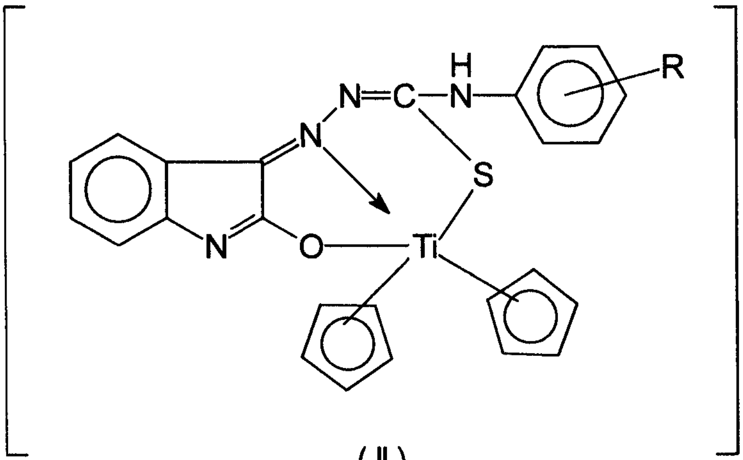

(II)

### Molluscicidal Activity

The molluscicidal activity of bis(cyclopentadienyl)titanium(IV) derivatives against snail ***Lymnaea acuminata*** was recorded. This snail is the intermediate host of ***Fosciola hepatica,*** which causes endemic ***fascioliosis*** in the cattle population of eastern U.P. (India). The effect of sublethal exposure of [$Cp_2$Ti(IPMET)] on acetylcholinesterase (AchE) activity in the nervous tissue of snails was studied. AChE is responsible for the termination of cholinergic impulses by the hydrolysis of acetylcholine released during synaptic transmission.

**Table 4**

Toxicity ($LC_{50}$) of different thiosemicarbazones of cyclopentadienyltitanium(IV) against *lymnaea acuminata*

| Periods | Compounds | $LC_{50}$ (w/v)mg/L | Limits LCL | UCL | Slope Value | t-ratio | g-value | Heterogeneity |
|---|---|---|---|---|---|---|---|---|
| 24 h | [$Cp_2$Ti(IPMT)] | 0.15 | 0.09 | 0.57 | 1.44±0.36 | 4.02 | 0.237 | 0.39 |
| | [$Cp_2$Ti(IPT)] | 0.12 | 0.09 | 0.23 | 2.26±0.52 | 4.31 | 0.206 | 0.25 |
| | [$Cp_2$Ti(IPMET)] | 0.098 | 0.06 | 0.21 | 1.50±0.32 | 4.65 | 0.177 | 0.24 |
| | [$Cp_2$Ti(IOMT)] | 0.12 | 0.09 | 0.20 | 2.79±0.60 | 4.58 | 0.182 | 0.32 |
| 48 h | | | | | | | | |
| | [$Cp_2$Ti(IPMT)] | 0.11 | 0.07 | 0.41 | 1.13±0.29 | 3.87 | 0.256 | 0.19 |
| | [$Cp_2$Ti(IPT)] | 0.08 | 0.07 | 0.13 | 1.94±0.44 | 4.42 | 0.19 | 0.21 |
| | [$Cp_2$Ti(IPMET)] | 0.07 | 0.05 | 0.16 | 1.21±0.28 | 4.30 | 0.208 | 0.17 |
| | [$Cp_2$Ti(IOMT)] | 0.10 | 0.08 | 0.19 | 1.82±0.44 | 4.08 | 0.231 | 0.30 |
| 72 h | | | | | | | | |
| | [$Cp_2$Ti(IPMT)] | 0.05 | 0.03 | 0.09 | 1.09±0.26 | 4.12 | 0.226 | 0.15 |
| | [$Cp_2$Ti(IPT)] | 0.05 | 0.05 | 0.07 | 2.02±0.45 | 4.88 | 0.161 | 0.21 |
| | [$Cp_2$Ti(IPMET)] | 0.03 | 0.02 | 0.04 | 1.28±0.26 | 4.85 | 0.163 | 0.15 |
| | [$Cp_2$Ti(IOMT)] | 0.06 | 0.05 | 0.07 | 1.9±0.41 | 4.63 | 0.179 | 0.19 |
| 96 h | | | | | | | | |
| | [$Cp_2$Ti(IPMT)] | 0.02 | 0.01 | 0.02 | 1.47±0.26 | 5.49 | 0.127 | 0.27 |
| | [$Cp_2$Ti(IPT)] | 0.04 | 0.03 | 0.04 | 2.48±0.42 | 5.91 | 0.11 | 0.39 |
| | [$Cp_2$Ti(IPMET)] | 0.01 | 0.01 | 0.02 | 1.44±0.26 | 5.29 | 0.137 | 0.47 |
| | [$Cp_2$Ti(IOMT)] | 0.04 | 0.03 | 0.05 | 1.95±0.40 | 4.80 | 0.166 | 0.24 |

Batches of 10 snails were exposed to four different concentrations of the above treatment. Morality was determined every 24 h. Each set of experiment was replicated six times.

Laboratory toxicity evaluation of all the four compounds was both dose dependent and time dependent. Toxicity of [$Cp_2$ Ti(IPMET)] ($LC_{50}$=0.098 mg/L for 24 h) was highest against ***L. acuminata*** (Table 4). It is clear that all four compounds are potent molluscicides and their toxicity is more pronounced with respect to other synthetic molluscicides /36/. The 96h $LC_{50}$ of *Mexacarbamates* : 1.7 mg/L, carbaryl : 4.4 mg/L,

Phorate: 15 mg/L, Formathion: 8.5 mg/L are higher than those of bis(cyclopentadienyl) derivatives of titanium(IV). The order of 48 h toxicity against ***L. acuminata*** is :[$Cp_2$Ti(IPMET)]> [$Cp_2$Ti(IPT)] > [$Cp_2$Ti(IOMT)] > [$Cp_2$Ti(IPMT)] (Fig. 1). The slope values were steep and results were found to be within 95% confidence limits of $LC_{50}$. Steep slope value of ldp line indicate that a small increase in the concentration of compound cause large mortality in snail. The t-ratio greater than 1.96 indicate that a regression is significant. Heterogeneity factor values less than 1.0 denote that in the replicate tests of random samples, the concentration response line would fall within 95% confidence limit and thus model fits the data adequately. The index of significance of potency estimation (g) indicates that the value of the mean is within the limits at all probability levels (90%, 95%, 99%) as it is less than 0.5. Sublethal treatment of [$Cp_2$Ti(IPMET)] caused significant change in the AChE activity in the nervous tissue of ***L. acuminata***. 40% and 80% of 24h $LC_{50}$ treatment caused a reduction in AChE activity up to 32% (0.35±0.022 μm'SH'hydrolysed/min/mg/protein) and 28.85% (0.101±0.007 μm'SH'hydrolysed/min/mg/protein) of control (0.35±0.022 μm 'SH' hydrolysed/min/mg/protein), respectively.

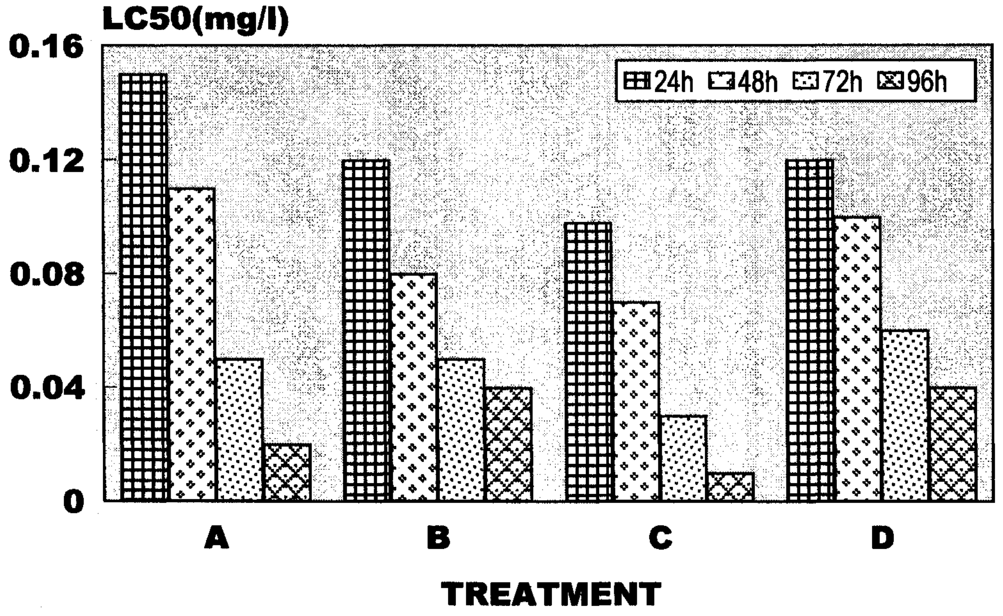

**Fig. 1:** Toxicity of different bis(cyclopentadienyl)titanium(IV) theiosemicarbazone derivatives against *Lymnaea acuminata*; A = [$Cp_2$Ti)IPMT)], B = [$Cp_2$Ti(IPT)], C = [$Cp_2$Ti(IPMET)], D = $Cp_2$Ti(IOMT)

It can be concluded from the present study that [$Cp_2$Ti(IPT)], [$Cp_2$Ti(IPMT)], [$Cp_2$Ti(IOMT)] and [$Cp_2$Ti(IPMET)] adversely affect the neurotransmission mechanism in the snail. These compounds are both metabolised in the snail body and transformed into more toxic form than their parent compounds or in due course of time *i.e.* from 24 h to 96 h exposure, there is an increase in concentration of the compound inside the snail body, which ultimately cause more mortality at higher exposure period.

### ACKNOWLEDGEMENT

The authors are thankful to Dr. D.K. Singh, Zoology Department, DDU Gorakhpur University, for his help during toxicity studies. One of the authors (GV) thanks CSIR, New Delhi for the award of JRF.

### REFERENCES

1. J.S. Casas, M.V. Castano, M.C. Cifuentes, A. Sanchez and J. Sordo, *Polyhedron*, **21**, 1651 (2002).
2. E. Labisbal, A. Sousa-Pedrares, A. Castineiras, J.K. Swearingen and D.X. West, *Polyhedron*, **21**, 1553 (2002).
3. J.K. Swearingen and D.X. West, *Transition Met. Chem.*, **26**, 252 (2001); **25**, 241 (2000).
4. M. Akbar Ali, A.H. Mirza, A.M.S. Hossain and M. Nazimuddin, *Polyhedron*, **20**, 1045 (2001).
5. D.X. West, A.E. Liberta, S.B. Padhye, R.C. Chikate, P.B. Sonawane, A.S. Kumbhar and R.G. Yerandi, *Coord. Chem. Rev.,* **123**, 49 (1993).
6. M. Akbar Ali and S.E. Livingstone, *Coord. Chem. Rev.*, **13**, 101 (1974) and ref. therein.
7. S. Padhye and G. B. Kauffman, *Coord. Chem. Rev.*, **63,** 127 (1985)
8. J.S. Casas, M.S. Garcia-Tasende and J. Sordo, *Coord. Chem. Rev*, **209**, 197 (2000).
9. H. Beraldo, R. Lima, L.R. Teixeira, A.A. Moura and D.X. West, *J. Mol. Struct*, **559**, 99 (2001).
10. F. Chen-jie, D. Chun-ying, H. Cheng, H. Gang and M. Qing-Jim, *New J. Chem*, **24**, 697 (2000).
11. D.X. West, S.B. Padhye and P.B. Sonawane, *Struct. Bonding*, **76,** 1 (1991).
12. M.J.M. Campbell, *Coord. Chem. Rev.*, **15**, 279 (1975).
13. R.W. Brockman, J.R. Thomson, M.J. Bell and H.E. Skippar, *Cancer Res.*, **16**, 167 (1956).
14. V.M. Leovac, V.I. Cesljevic, G. Argay, A. Kalman and B. Riber, *J. Coord. Chem.*, **34**, 357 (1995).
15. D. Singh and R.V. Singh, *J. Inorg. Biochem.*, **15**, 227 (1993).
16. M. Mohan, A. Agrawal and N.K. Jha, *J. Inorg. Biochem*, **34**, 41 (1988).
17. J.S. Casas, A. Castineiras, A. Sanchez, J. Sordo, A. Vazquez-Lopez, M.C. Rodriguez-Arguelles and U. Russ, *Inorg. Chim. Acta*, **61**, 221 (1994).
18. D.X. West, H. Gebremdhin, T.J. Romack and A.E. Liberta, *Transition Met. Chem.*, **19,** 426 (1994).
19. S.N. Shetti, A.S. Murthy and G.L. Tenbe, *Transition Met. Chem.*, **18**, 467 (1993).
20. X. Shen, Y. Xie and H. Geng, *Synth. React. Inorg. Met.-Org. Chem.*, **24,** 1351 (1994).
21. M.C. Miller III, K.F. Bastow, C.N. Stineman, J.R. Vance, S.C. Song, D.X. West, I.H. Hall, *Archiv der Pharmazie*, **331**, 121 (1998).
22. E.J. Blanz Jr. and F.A. French, *Cancer Res.*, **28**, 2419 (1968).
23. R.L. Thompson, S.A. Milton, J.E. Officer and G.H. Hitchings, *J. Immunol*, **70**, 229 (1953).
24. D.J. Bauer, *Brit. J. Exp. Path.*, **36**, 105 (1956).
25. D.J. Bauer and P.W. Sadler, *Brit. J. Pharmacol*, **15**, 101 (1960).
26. G.A. Bain, D.X. West, J. Krejci, J.V. Martinez, S.H. Ortega and R.A. Toscano, *Polyhedron*, **16**, 855 (1997).

27. E. Labisbal, A. Sousa, A. Castineiras, J.A. Garcia-Vazquez, J. Romero and D.X. West, *Polyhedron*, **19**, 1255 (2000).
28. M.C. Rodriguez-Arguelles, A. Sanchez, M.B. Ferrari, G.G. Fava, C. Pelizzi, G. Pelosi, A. Albertini, P. Lunghi and S. Pinelli, *J. Inorg. Biochem.*, **73**, 7 (1999).
29. J.S. Casas, E.E. Castellano, M.S. Garcia Tasende, A. Sanchez and J. Sordo, *Inorg. Chim. Acta*, **304**, 283 (2000).
30. J.S. Casas, A. Castinerias, M.C. Rodriguez-Arguelles, A. Sanchez, J. Sordo, A. Vazquez-Lopez and E.N. Vazquez-Lopez, *J. Chem. Soc. Dalton Trans.*, **2000**, 4056.
31. A.I. Vogel, *A Text Book of Practical Organic Chemistry*, Longman's Green, London, 1948.
32. G. Wilkinson and J.M. Birmingham, *J. Am. Chem. Soc.*, **76**, 4281 (1954).
33. S.K. Sengupta, O.P. Pandey, M.K. Mishra and C.M. Tripathi, *Bioinorg. Chem. and Applications*, In press (2003).
34. R.M. Russell, J.L. Robertson and N.E. Sevin, POLO: A new computer program for probit analysis. *Bull. Entomol. Soc. Am.*, **23**, 209 (1977).
35. G.L. Ellaman, K.D. Courtney, V. Andres Jr. and R.M. Featherstone, *Biochem. Pharmacol*, **7**, 88 (1961).
36. D.K. Singh and R.A. Agrawal, *Arch. Environ. Contam. Toxicol*, **12**, 483 (1983).
37. O.H. Lowery, N.J. Rosebrough, A.L. Farr and R.J. Randall, *J. Biol. Chem.*, **193**, 265 (1951).
38. S.K. Sengupta, O.P. Pandey, B.K. Srivastava and V.K .Sharma, *Transition Met. Chem.*, **23**, 349 (1998).
39. U.K. Pandey, O.P. Pandey, S.K. Sengupta and S.C. Tripathi, *Polyhedron*, **6**, 1611, (1987).
40. A. Rai, S.K. Sengupta and O.P. Pandey, *Indian J. Chem.*, **39A**, 1198 (2000).
41. R. Rai, K.D. Mishra, O.P. Pandey and S.K. Sengupta, *Polyhedron*, **11**, 123 (1992).

